# Proactive familial cancer risk assessment: a service development study in UK primary care

**DOI:** 10.3399/BJGPO.2023.0076

**Published:** 2023-12-13

**Authors:** Abdul Rahman Badran, Alice Youngs, Andrea Forman, Marisa Elms, Lai-Lai Chang, Fiyaz Lebbe, Adam Reekie, John Short, Min Theik Hlaing, Emma Watts, Deborah Hipps, Katie Snape

**Affiliations:** 1 South West Thames Centre for Genomics, St George’s University Hospitals NHS Foundation Trust, London, UK; 2 Fieldway Medical Centre, Danebury, New Addington, Croydon, UK; 3 West Barnes Surgery, West Barnes Lane, London, UK; 4 Shere Surgery, Gomshall Lane, Guildford, UK; 5 The Exchange Surgery, Gracefield Gardens, London, UK

**Keywords:** primary health care, medical history taking, early detection of cancer, general practitioners

## Abstract

**Background:**

Family history assessment can identify individuals above population-risk for cancer to enable targeted Screening, Prevention, and Early Detection (SPED). Family History Questionnaire Service (FHQS) is a resource-efficient patient-facing online tool to facilitate this. In the UK, cancer risk assessment is usually only offered to concerned individuals proactively self-presenting to their GP, leading to inequity in accessing SPED in the community.

**Aim:**

To improve access to community cancer genetic risk assessment and explore barriers to uptake.

**Design & setting:**

Service development project of a digital pathway using the FHQS for cancer risk assessment across four general practices within the clinical remit of the South West Thames Centre for Genomics (SWTCG).

**Method:**

3100 individuals aged 38–50 years were invited to complete the FHQS through either text message or email. A random selection of 100 non-responders were contacted to determine barriers to uptake.

**Results:**

In total, *n* = 304/3100 (10%) registered for the FHQS. Responders were more likely to be British (63% vs 47%, *P*<0.001), speak English as their main language (92% vs 76%, *P*<0.001), and not require an interpreter (99.6% vs 94.9%, *P* = 0.001). Of 304 responders, 158 (52%) were automatically identified as at population-risk without full family history review. Of the remaining 146 responders, 52 (36%) required either additional screening referral (*n* = 23), genetics referral (*n* = 15), and/or advice to relatives (n = 18). Of 100 non-responders contacted, eight had incorrect contact details and 53 were contactable. Reasons for not responding included not receiving invitation details (*n* = 26), losing the invitation (*n* = 5), or forgetting (*n* = 4).

**Conclusion:**

The FHQS can be used as part of a low-resource primary care pathway to identify individuals in the community above population-risk for cancer requiring action. This study highlighted barriers to uptake requiring consideration to maximise impact and minimise inequity.

## How this fits in

Family history assessment for cancer can be used to identify individuals who have an inherited predisposition to cancer development, providing the opportunity for targeted intervention. This study demonstrated the use of an online patient-facing tool for family history assessment in the primary care setting, as part of a novel proactive low-resource pathway. Barriers to uptake are highlighted and require consideration to maximise impact and minimise inequity.

## Introduction

Screening for Prevention and Early Detection (SPED) of cancer can be cost-effective and preferable to other therapeutic interventions.^1^ This is achieved through increased detection of cancer at a curable stage and decreasing the likelihood of cancer development, for example surveillance for identification and treatment of pre-cancerous polyps. The benefits of additional screening and early treatments are well-established in familial colorectal and breast cancer.^2–5^

A monogenic predisposition is estimated to contribute to up to 10% of cancer development, with an even larger proportion being polygenic.^6–8^ In a California-based population study, around 20% of individuals without a personal history of cancer were found to be above population-risk for breast, ovarian, endometrial, prostate, or colorectal cancer.^9^ It is estimated that a general practice with a list size of 2000 patients would have 30–40 patients with a family history of colorectal, breast, ovarian, or uterine cancer, requiring management consideration.^10^ Identifying those with increased cancer susceptibility enables efficient targeting of interventions to those above-population risk, for example lifestyle advice, prophylactic medications, screening investigations, genetic testing, and surgical procedures. Family history assessment in primary care is a low-cost intervention, offering opportunities to identify individuals above population-risk for cancer, and eligible for interventions prior to national screening programme eligibility.^11–14^ Moreover, being aware of familial cancer risk does not appear to increase psychological distress in patients.^15,16^

Family history risk assessment tools aid collection and use of cancer family history in primary care.^17^ These perform well against gold standard genetic interviews, but there is a lack of general cancer family history assessment tools suitable for primary care use. The Cancer Genetics Unit of South-West Thames Centre for Genomics (SWTCG) at St George’s Hospital developed the Family History Questionnaire Service (FHQS).^18^ This is an online patient-facing family history data collection software application, streamlining assessment of cancer genetic susceptibility across all tumour types for competent and standardised cancer family history assessment, which is efficient, cost-effective, and scalable.

Based on the authors’ experience of UK general practice, cancer risk assessment is usually only offered to individuals self-presenting to their GP with concerns about their family history, which can lead to inequity in access to SPED interventions. In 2022, the National Institute for Health and Care Excellence (NICE) withdrew a statement recommending breast cancer risk-assessment in primary care only when women present with concerns, shifting to more proactive assessment.^19^ Proactive cancer risk-assessment using a risk-assessment tool has previously been trialled in primary care, delivered via postal questionnaire.^20^ This focused on familial breast cancer risk-assessment, demonstrating accurate identification of women above population-risk. Responders were predominantly of White ethnicity (98%) with a large proportion of university-educated individuals (40%).

The aim of this study was to improve regional access to cancer genetic risk assessment in the community and determine barriers to uptake. A proactive digital pathway was developed using the FHQS and piloted as a service development project at four general practices to assess impact on identification of individuals above population-risk for cancer and to determine resource implications.

## Method

### Practice and patient recruitment

**Figure 1.**
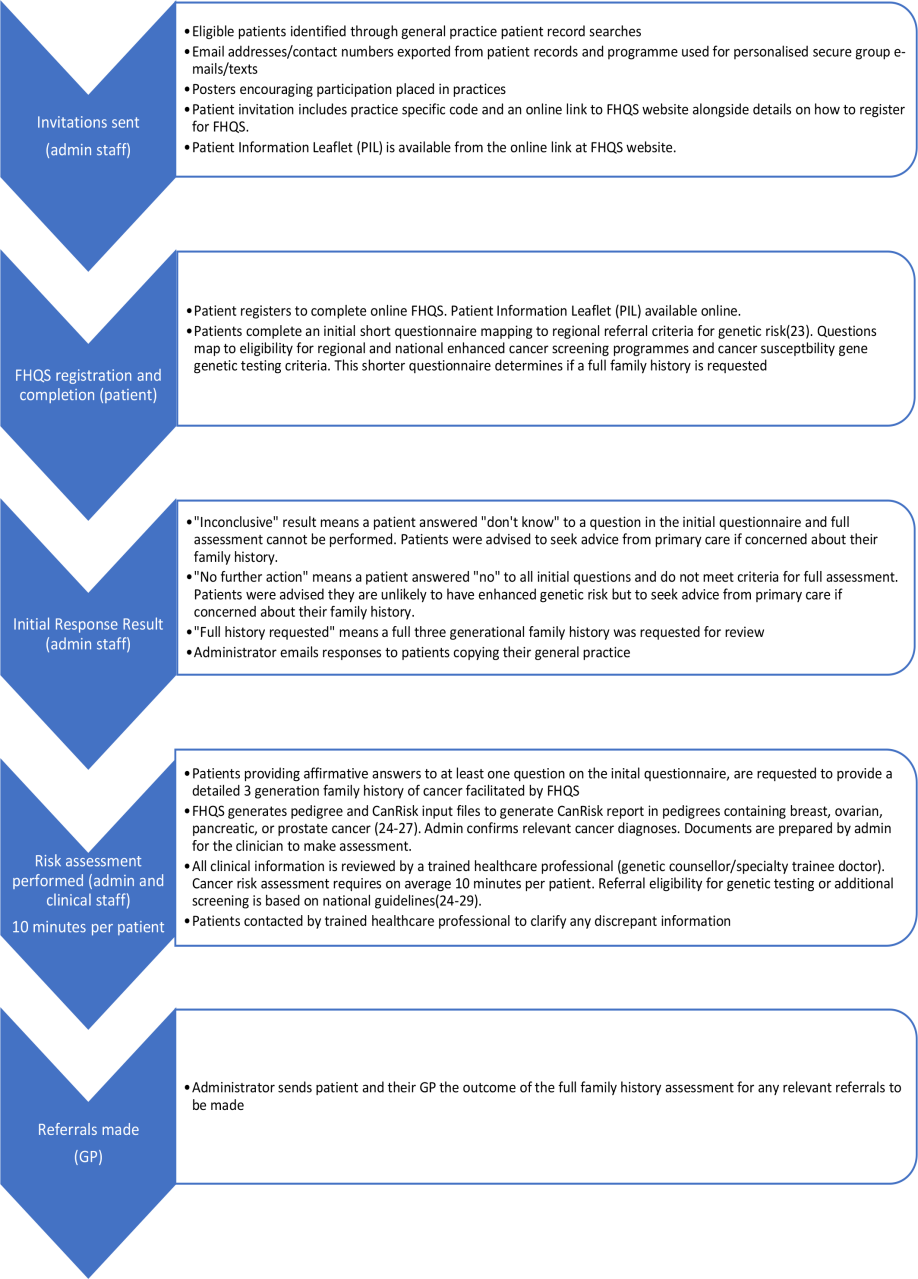
FHQS pathway for invitation for assessment and onwards referral.

General practices within the remit of SWTCG were invited to participate via email communication as part of a clinical service development project to improve access to cancer risk assessment. The four general practices enrolled were chosen in areas across the deprivation spectrum in the UK according to Official for National Statistics data.^21^ Patients aged between 38–50 (see full recruitment details below) registered at four general practices, were invited to complete the FHQS between January 2022 and January 2023. One of the practices (Practice C) is in a rural area whereas the other practices are in urban areas. Figure 1 is a summary of the processes followed for patient invitation and risk-assessment.

### Patient pathway

General practice administrative staff identified eligible patients for this project through EMIS patient record searches of eligible age ranges and invited them to complete the FHQS by email or text message. Patient confidentiality was maintained through anonymising patient data and secure communications between genetics department staff and general practices.

In order to iteratively review and improve the patient pathway and resource impact on practice staff before wider roll-out, recruitment was initially restricted to a single pilot site, Practice A. Recruitment at this practice was through two different patient cohort invitations. The initial recruitment commenced January 2022 and then extended recruitment from September 2022:

#### Cohort 1: January 2022

An initial cohort, of all patients aged 40–50, who had not previously completed an NHS health check were invited by text message to complete their NHS Health Check alongside the FHQS. This pathway efficiently made use of allocated resource for the established NHS health check service, by ‘adding’ the cancer genetic risk assessment alongside the NHS Health Check. Further contact was made with non-responders to encourage participation.

#### Cohort 2: September 2022

Once the administrative and clinical infrastructure was embedded for this smaller cohort, and a review of the resource implications for Practice A had been undertaken, text invitations were extended to all patients in the age range 38–40 years with the aim of capturing at risk females prior to eligibility for additional mammographic screening.

In addition, from September 2022, to try to boost recruitment, the practice highlighted the FHQS invitation to all patients aged between 38–50 years presenting to Practice A for other reasons. To encourage engagement, posters were placed in the waiting room, a message was displayed on a TV screen, and patient information leaflets were provided at reception. Individuals having remote consultations were sent the FHQS invitation letter by email. This opportunistic approach would have included both Cohort 1 and 2 above and, additionally, patients aged 40–50 who had completed the NHS Health Check prior to Cohort 1 invitations and had not previously been invited by text message.

Practices B–D were subsequently enrolled from July 2022 using a tighter age range of 38–45 years as this closely spans the starting eligibility age for moderate-risk breast cancer screening. All practices used a combination of text and email invitations to eligible patients, with some additionally encouraging participation via other means (for example local newsletter) resulting in minor differences in the exact engagement format. A random selection of 100 non-responders at Practice D, in the most deprived area (see Table 1), were called to determine barriers to uptake.

**Table 1.** Comparison of invited patients’ characteristics among the general practices

Practice	Index of Multiple Deprivation decile (1 is most deprived)	Education and training decile (1 is most deprived)	Health deprivation decile (1 is most deprived)	Female gender, % (*n/N*)	British nationality, % (*n/N*)	English main language, % (*n/N*)	Interpreter required, % (*n/N*)
**A**	6	9	7	44 (313/709)	27 (79/295)	61 (177/291)	9 (65/709)
**B**	10	10	10	44 (402/913)	42 (351/828)	72 (436/609)	0 (0/913)
**C**	8	6	10	54 (293/541)	82 (432/530)	95 (446/468)	0.4 (2/541)
**D**	2	2	3	50 (472/937)	43 (345/805)	79 (566/714)	4 (34/937)
**Total**				48 (1480/3100)	49 (1207/2458)	78 (1625/2082)	5 (101/2187)

### Family History Questionnaire Service (FHQS)

The FHQS is an online patient-facing cancer family history data collection application developed by SWTCG.^18,22^ This collects the same information as the previously used paper family history questionnaire used by SWTCG, with the addition of initial questions ensuring patients meet regional criteria for full family history assessment.^23^

FHQS is designed to request the following information to determine possible genetic cancer risk:

- Age, type, and number of any tumour/cancer diagnoses in the proband and specific relatives (grandchildren, children, siblings, parents, aunts and uncles, grandparents). Other affected relatives can be added on a case by case basis;
- Sex, gender, and ethnicity of the proband.

FHQS is an online branching questionnaire that personalises the questions asked to previous responses.

Patient volunteers were involved in developing information leaflets, which were made available at online FHQS registration. Patients register and complete the FHQS online using a service code unique to their general practice, and consent for their data to be used for cancer risk analysis. Patients were contacted to clarify any conflicting information provided. For each proband, FHQS generates a PDF pedigree document (a visual representation of the family history with ages and tumour diagnoses), a pdf summary of all affected family members and a CanRisk input file, which can be inputted into CanRisk to generate a CanRisk report in pedigrees containing breast, ovarian, pancreatic, or prostate cancer. All clinical information was reviewed by a trained healthcare professional (genetic counsellor or specialty trainee) for risk assessment with the outcome communicated to the patient’s GP for actioning.

### Breast and ovarian cancer risk assessment

CanRisk is a web interface to the BOADICEA risk prediction model for calculating risks of breast and ovarian cancer in women, and is supported for clinical use by NICE guidelines.^24–27^ CanRisk generates a report with a risk-category for breast cancer likelihood (near-population, moderate, high) and likelihood of a predisposing pathogenic variant. For high-risk patients, diagnoses were confirmed through the National Cancer Registration and Analysis Service for bilateral breast cancer, breast cancer under the age of 40, and ovarian cancer as inaccurate reporting significantly impacts risk assessment.

### Colorectal cancer risk assessment

Colorectal cancer risk was determined based on British Society of Gastroenterology guidelines for hereditary colorectal cancer management, categorised as average, moderate, or high.^28^

### Other cancer risk assessment

Assessment using National Genomic Test Directory eligibility criteria for germline genetic testing was made for pedigrees containing other cancers, alongside an assessment of whether further investigations or examinations were required.^29^

### Risk assessment outcomes

Secondary care referrals for additional mammography screening from age 40–49 years were recommended for moderate-risk breast cancer. Secondary care referrals for colonoscopy at the age of 55 years were recommended for moderate-risk colorectal cancer. Individuals at high-risk for breast or colorectal cancer, or meeting NHS National Genomic Test Directory criteria for genetic testing, were recommended for genetic referral.^29^ Relatives potentially eligible for testing or additional screening were highlighted to inform the family that they could request their own assessment. Assessment outcomes were sent to general practices for actioning referrals.

### Statistical analysis

Descriptive and statistical analysis was performed using Microsoft Excel and SPSS (version 29). Χ^2^ tests were used to assess for relation between response rate and gender, nationality, main spoken language, and requirement of an interpreter. These demographics are routinely collected by general practices and were deemed a priori as important factors that can influence response, as well as important demographics to consider when determining inequity in engagement across the invited population.

## Results

A breakdown summary of the invited patients’ characteristics for each general practice is presented in Table 1, and individual patients’ responses for each general practice in Table 2. Summary flowcharts for individual practices are found in the supplementary materials.

**Table 2.** Comparison of responses among the general practices

Practice	Invited, *n*	Completed*, n* (%)	Screened out, *n*	Full family history reviewed, *n*	Proportion of full family historyreviewed requiring action, % (*n/N*)
**A**	709	25 (4)	12	13	38 (5/13)
**B**	913	78 (9)	36	42	40 (17/42)
**C**	541	116 (21)	61	55	36 (20/55)
**D**	937	85 (9)	49	36	28 (10/36)
**Total**	3100	304 (10)	109	146	36 (52/146)

**Figure 2.**
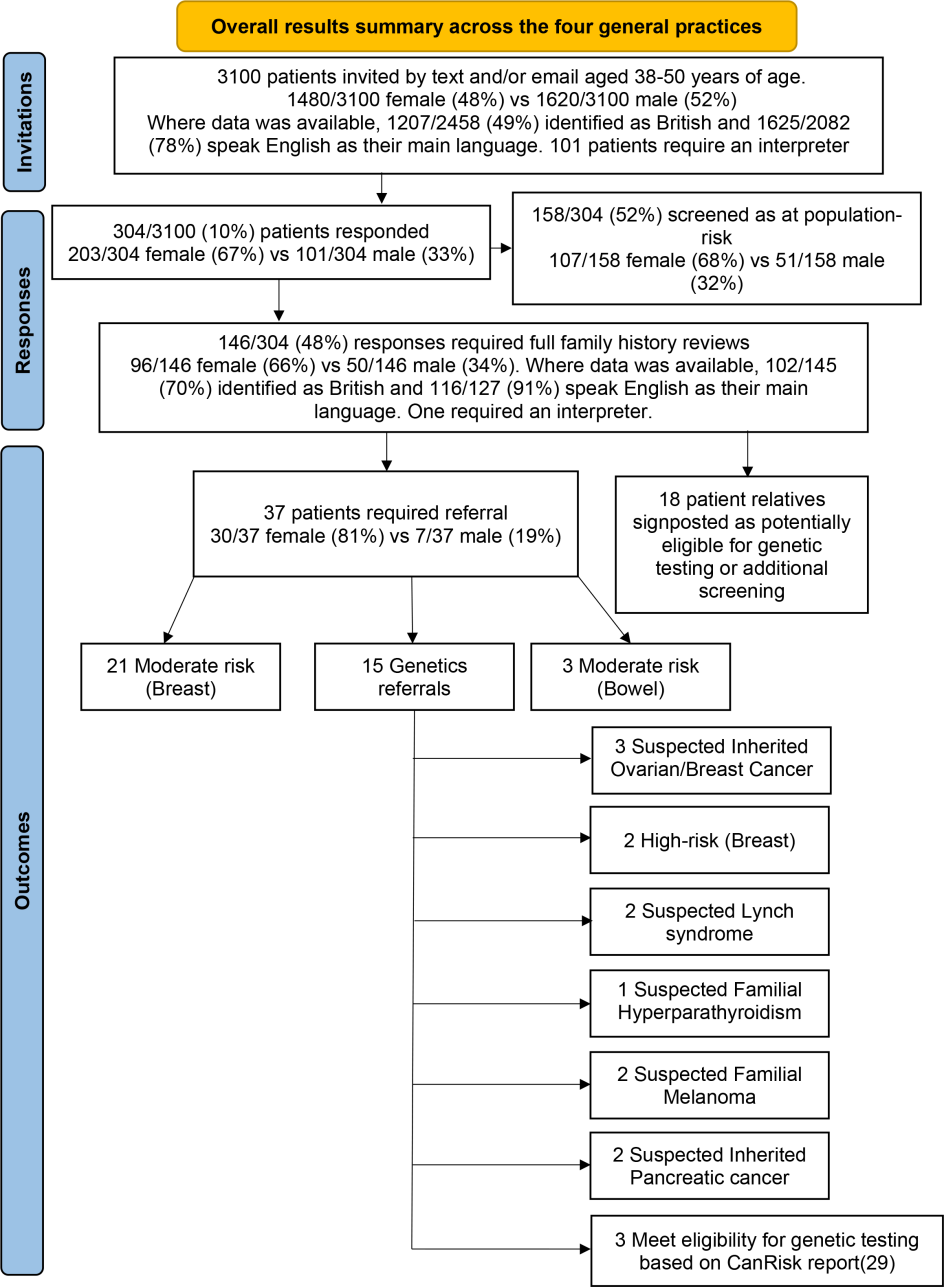
Summary flowchart of responses across the four general practices. Two of the patients requiring referral required more than one referral.

Across four general practices, 3100 patients were identified aged 38–50 years (average ±SD, 41.5 ±2.7 years) from patient record searches. Where known, most patients were male (*n* = 1620/3100, 52%) and identified as non-British (*n* = 1251/2458, 51%), with the majority speaking English as their main language (*n* = 1625/2082, 78%). A minority of these patients require an interpreter (*n* = 101/2187, 5%). Figure 2 is a response summary flowchart.

Of 3100 patients invited, 304 (10%) completed the FHQS. Responders were of similar age to non-responders (41.6 ±2.4 years vs 41.5 ±2.7 years, respectively). Patients completing the FHQS were more likely to be female than non-responders (67% *vs* 48%, Χ^2^ statistic 48.9, *P*<0.001). Responders were also more likely to be British (63% vs 47%, Χ^2^ statistic 28, *P*<0.001), speak English as their main language (92% *vs* 76%, Χ^2^ statistic 30.4, *P*<0.001) and not require an interpreter (99.6% vs 94.9%, Χ^2^ statistic 10.4, *P* = 0.001). Practice A with the lowest proportion of British (27%) and English-speaking (61%) individuals invited had the lowest response rate (4%), whereas Practice C with the highest proportion of British (82%) and English-speaking (95%) individuals invited had the highest response rate (21%). Of note, given females were more likely to be complete the FHQS; practice C with the highest response rate, also had the highest proportion of females (54%). Most responses (*n* = 158/304, 52%) were automatically categorised as at population-risk through FHQS initial screening questions for assessment eligibility. The remaining 146 (48%) responses required full family history review, with responders largely female (*n* = 99/146, 68%), and where known, the majority identified as British (*n* = 102/145, 70%) and speak English as their main language (*n* = 116/127, 91%). Only one responder needed an interpreter.

Of 146 responses requiring full family history review, 37 (25%) required either referral to genetics or for additional screening, with two requiring more than one referral. Most referrals were due to moderate-risk for breast cancer (*n* = 21/37, 57%), and only three referrals due to moderate-risk for bowel cancer. Genetics referrals were indicated for various reasons and are summarised in Figure 2. On full family history review, 18 patients had relatives potentially eligible for genetic testing or additional screening, and were highlighted in the risk assessment outcome.

Of the 709 patients invited to complete the FHQS from Practice A, Cohort 1 consisted of 289 patients, and cohort 2 of 420 patients. These cohorts had similar response rates (*n* = 12 vs 13). The numbers are too small to draw further significant insights from these data.

### Opportunistic invitations at practice A

**Figure 3.**
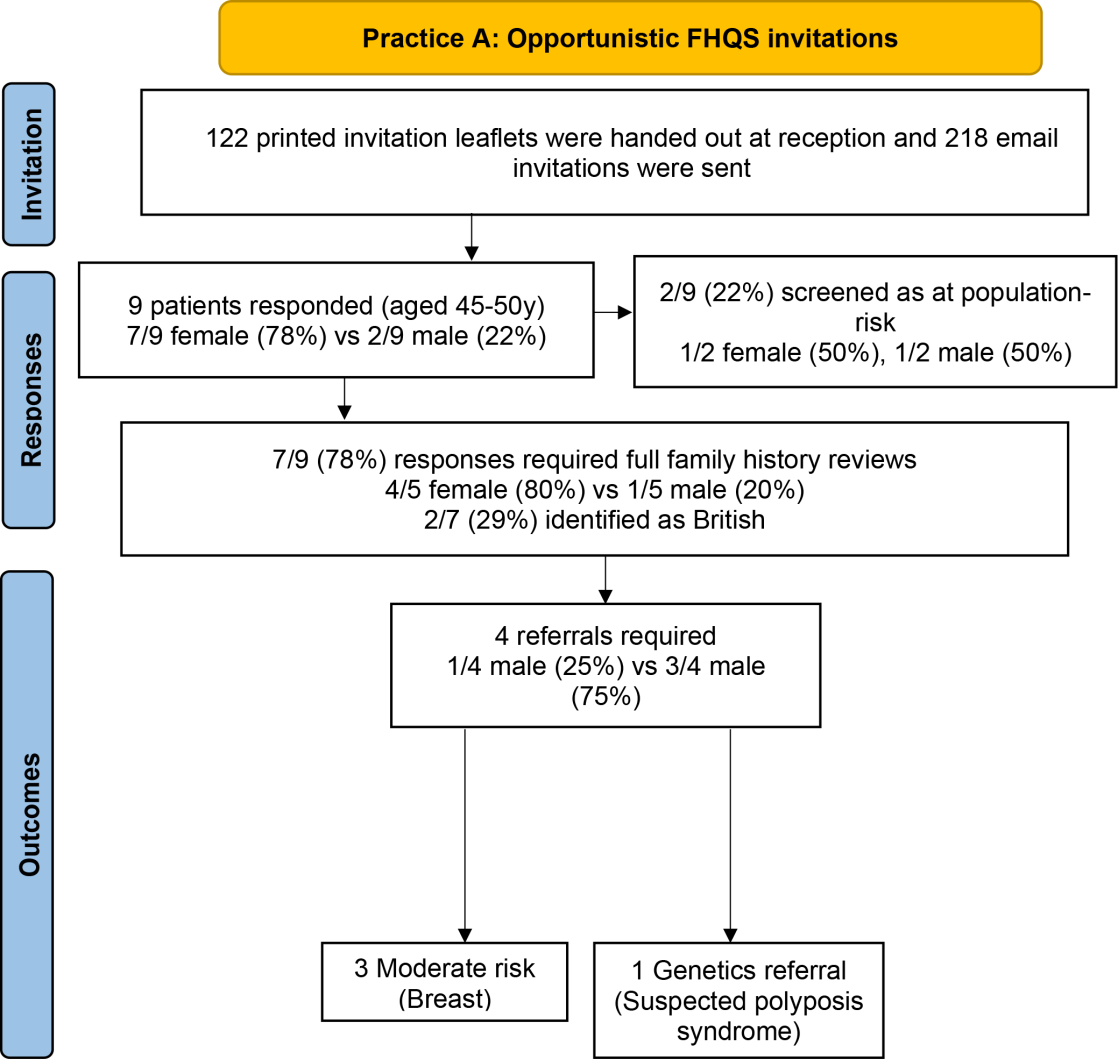
Summary results flowchart for opportunistic FHQS invitations at Practice A.

Over 122 invitation leaflets were collected by patients and 218 email invitations were sent. Nine patients aged 45–50 years who had not received a prior invitation responded, and were largely female (*n* = 7/9, 78%). Of these, 2 responses (22%) were automatically categorised as at population-risk through initial screening answers. The remaining *n* = 7/9 (78%) responses required full family history review: of these, *n* = 1/7 (14%) required a referral to genetics due to suspected polyposis syndrome; and *n* = 3/7 (43%) required referrals for additional screening due to moderate-risk for breast cancer. Only two of the seven responders who provided ethnicity information identified as White British. Figure 3 is a response summary flowchart.

**Figure 4.**
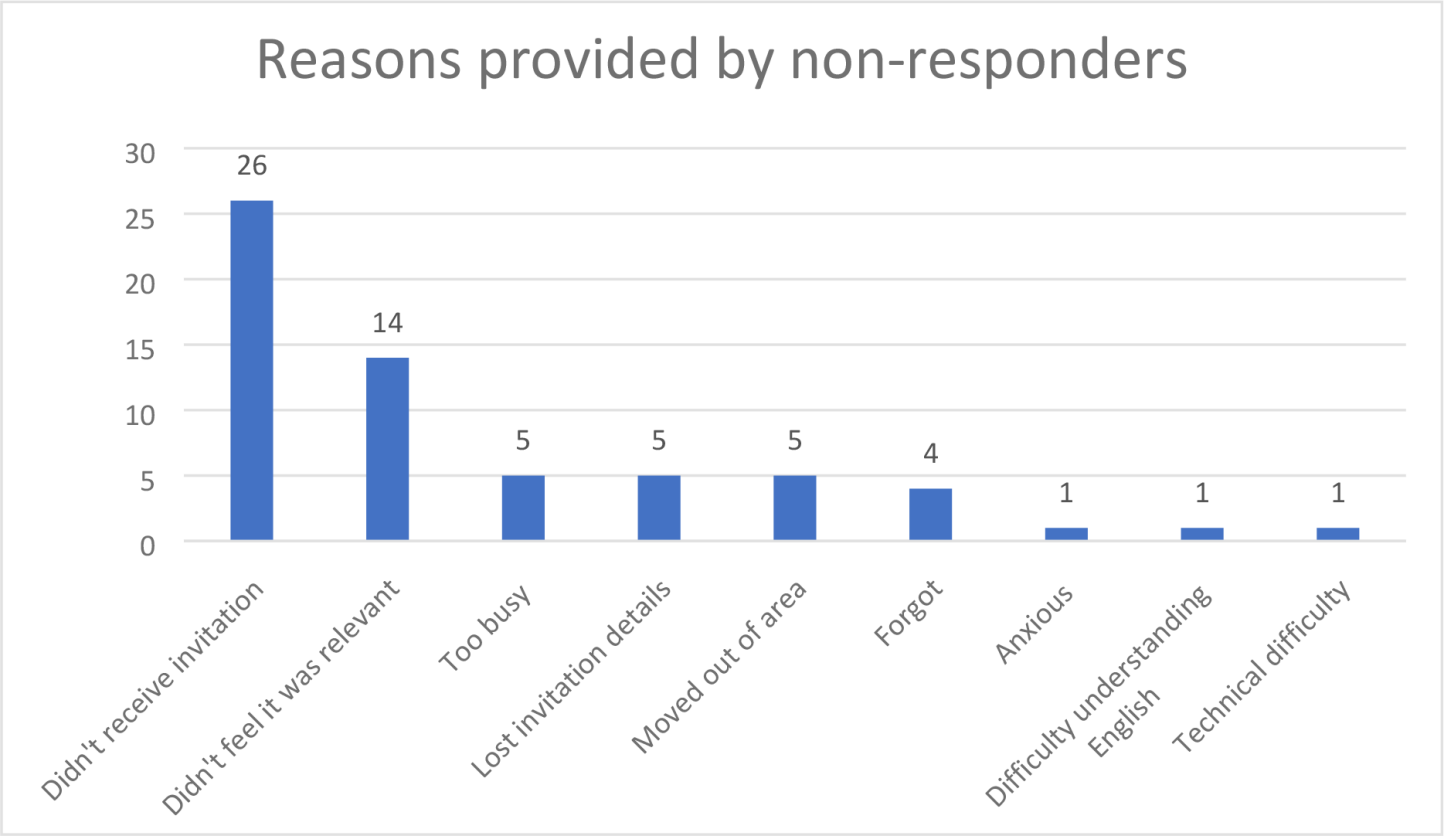
Reasons provided for not completing the FHQS by contactable non-responders (*n* = 53; non-responders could give more than one reason)

Seven responses were during a period where opportunistic invitations were concurrent with the second stage of the pilot at Practice A, with responders having potentially been opportunistically invited in addition to batch invitation. These responses were included in the Practice A Extended 38–40 years cohort response results, unless the responders were older than 40.

### Barriers to uptake

Non-responders at the initial stage of the Practice A pilot were contacted to encourage engagement, where it was identified *n* = 95/289 (33%) patients had incorrect contact details on their records.

A reminder text message sent to non-responders at Practice D 2 months after the initial invite resulted in 12 additional responses. A random selection of 100 non-responders at Practice D were contacted to determine barriers to uptake. There was a slight preponderance of females (52, 52%) in this sample, and where information was available, the sample was largely non-British (*n* = 50/85, 59%), with most speaking English as their main language (*n* = 60/82, 73%). 8/100 (8%) non-responders could not be contacted due to the contact number being incorrect. Of the 100 invited non-responders, 39 did not respond; 8 could not be contacted due to the contact number being incorrect; and 53 gave a reason for non-engagement, summarised in Figure 4 (responders could give more than one reason). Of these, *n* = 9 (17%) contactable non-responders eventually completed the FHQS, with one identified as moderate-risk breast cancer.

## Discussion

### Summary

To achieve the aim of improving access to cancer genetic risk assessment in the community, this study implemented and analysed a novel digital clinical pathway for proactive cancer family history assessment of all tumour types in both genders in UK primary care. As this project was a service development project designed to extend clinical assessment for cancer genetic risk in primary care, there are minor differences in design and implementation reflecting real-world differences between the practices involved and iterative improvements in pathway design to maximise clinical benefit to patients.

Of 3100 patients receiving immediate batch invitations, 304 (10%) engaged with an online patient-facing tool to assess familial cancer risk, with *n* = 37/304 (12%) requiring referrals. These patients had not previously self-presented to their GP asking for cancer risk assessment. Sending batch text or email invitations to patients can be undertaken by admin staff to set rules (for example, age limits) against a practice patient cohort and is a low resource mechanism for invitation.

General practices enrolled were in areas across the deprivation scale (2^nd^–10^th^ decile), though this did not correlate with response rate. Individuals identifying as non-British and those speaking a language other than English as their main language were significantly underrepresented in the proportion responding and engaging compared to the cohort invited overall. Differences in population characteristics and methods of patient engagement likely contributed to the discrepancy in response rates (3–21%) between general practices. The general practices were also enrolled at different time periods. Although these differences affect this study’s ability to draw comparisons, the authors feel these are pragmatic differences due to individual practice differences allowing real-word impact to be more discernible.

Practice A, which had the lowest response rate (3–4%), serves a population with the highest proportion of individuals identifying as non-British, speaking a language other than English as their main language, and requiring an interpreter. Anecdotally, patients are often transient and migratory, consistent with *n* = 95/289 (33%) of the invited population in the initial stage of the Practice A pilot being found to have incorrect contact details. Opportunistic invitations were more resource-demanding than batch invitations, with a similar yield in identifying above population-risk individuals.

Analysis of barriers to uptake at Practice D suggest practical factors such as incorrect contact details, failure to receive invitation, being busy, or forgetting are the biggest hurdles to responding. This study’s data suggested a reminder invitation can result in additional responses. Although almost half of contactable non-responders reported not receiving the invitation, their contact details were correct. This suggests a potential benefit in contacting patients through various means to ensure receipt, with reminders. A small proportion (*n* = 8/100, 8%) of non-responders contacted had an incorrect contact number, indicating a need to ensure correct and up-to-date contact details in records to maximise impact.

The lower response rates from those registered as non-British, those with English as a second language, and those requiring an interpreter confirmed that additional methods of engagement are required to ensure equity of access. A low resource pathway for a proportion of patients should allow diversion of additional resource to improve engagement for others who require more support to complete assessments.

### Strengths and limitations

Although self-reported family history is reliable in first-degree relatives of individuals with ovarian and breast cancer, reliability from other relatives and about other cancers is unclear and may be affected by recall bias.^30,31^ There is potentially also an inherent inequity in individuals knowing their family history according to ethnicity and socioeconomic status.^32^ It is possible that the digital online format of the FHQS may have hindered participation from individuals without the means to engage online, however the authors did not receive any feedback from non-responders, patients, or general practices to this effect.

As the initial stage of the Practice A pilot was concurrent with the NHS health check, individuals who already completed the NHS health check were excluded, resulting in a potentially less engaged population being invited to complete the FHQS. Although this could contribute to a lower response rate, the rate was similar to the second stage of the Practice A pilot. Opportunistic invitations following batch invites may have impacted apparent yield as it is conceivable that interested individuals who were already invited would have already responded.

In this pilot, all family histories were reviewed by a trained healthcare professional. This enabled analysis of the number and type of histories received in this proactive approach. If embedded in routine clinical practice, the role of the GP would be to review a patient-provided family history and decide if onwards referral is required for further assessment. There are existing tools to support GPs with this decisionmaking.^33,34^ FHQS enables collection and presentation of family history data to the GP in a much more streamlined manner than current practice. High quality family history data provided by FHQS to accompany GP referrals into secondary and tertiary care would significantly improve current referral pathways. Obtaining this information at time of referral, in combination with digitalised data and administrators to generate documents relevant for risk assessment, would enable secondary and tertiary care healthcare professionals to complete genetic risk assessment in a more time-efficient and streamlined manner. Reducing inappropriate referrals from primary care into secondary and tertiary care service and streamlining risk assessment may offset any increase in appropriate and necessary referrals. Further work is underway to analyse the impact of this pathway within the authors’ region through the development of virtual family history clinics supported by regional genetics services.

### Comparison with existing literature

Proactive family history assessment for cancer has previously been studied in primary care by Qureshi and colleagues, though the focus was on breast cancer in women aged 30–60 years, missing the opportunity to capture a significant proportion of individuals above population-risk for other cancers.^20^ Moreover, family history assessment was using postal surveys and in a largely White population. Although response rate was higher than the current study (16% vs 10%), additional resources required for postal survey compared to online assessment make implementation and scalability more difficult. In Qureshi *et al*, 11.4% of the women responding were at increased risk of breast cancer, similar to this study’s results (11.3%). Their study also assessed opportunistic assessment but determined this to be sub-optimal, leading to fewer women identified at increased risk of breast cancer. This study highlighted barriers to uptake in primary care requiring consideration to maximise impact and minimise inequity. Furthermore, the responses in this study being predominantly from females despite a majority male population being invited is consistent with a population-based study suggesting males are less likely to report family history of cancer.^35^

### Implications for future practice and research

This study demonstrated the ability of a novel clinical pathway to identify individuals above population-risk for cancer in the community. This novel clinical pathway highlights the clinical impact of proactively collecting and acting on family history of cancer in primary care. Increasing engagement and comprehensively collecting family history of cancer proactively will identify at-risk patients earlier for screening and preventative interventions. However, additional work and resource is required to improve pathways to support minority ethnic groups and those with English as a second language. Identification of more genetically at-risk patients in primary care will require support from healthcare professionals trained in cancer genetics risk assessment in secondary and tertiary care. The development of regional virtual family history clinics to support primary care with proactive community ascertainment and onwards referral pathways is a potential model for scalable expansion of this service. The authors are undertaking a pilot of this model in their region alongside health economic analyses to assess the cost-benefit of this approach.

Assessment of longer-term outcomes of those referred via this pathway requires a study on a longer time scale. Moreover, the ideal interval for updating a patient’s risk-assessment needs to be determined as family history changes. Considering the barriers to uptake highlighted in this study and the variable response rates among the diverse practice populations invited, evaluation of targeted and tailored efforts is needed to improve engagement and minimise inequity. Other future considerations for the pathway include more resource-intensive invitation methods (mail and telephone call) and translation of the FHQS and invitation material into other languages.

## Data

The dataset relied on in this article is available from the corresponding author on reasonable request.
